# Pattern, Clinical Characteristics, and Outcome of Meningitis among HIV-Infected Adults Admitted in a Tertiary Hospital in North Western Tanzania: A Cross-Sectional Study

**DOI:** 10.1155/2016/6573672

**Published:** 2016-08-29

**Authors:** Matobogolo M. Boaz, Samuel Kalluvya, Jennifer A. Downs, Bonaventura C. T. Mpondo, Stephen E. Mshana

**Affiliations:** ^1^Department of Medicine, College of Health Sciences, The University of Dodoma, Dodoma, Tanzania; ^2^Department of Medicine, Weill Bugando School of Medicine, Mwanza, Tanzania; ^3^Department of Medicine, Bugando Medical Centre, Mwanza, Tanzania; ^4^Center for Global Health, Department of Medicine, Weill Cornell Medical College, New York, NY, USA; ^5^Department of Microbiology/Immunology, Weill Bugando School of Medicine, Mwanza, Tanzania

## Abstract

*Background.* Limited information exists on the etiologies, clinical characteristics, and outcomes of meningitis among HIV-infected patients in Africa. We conducted a study to determine the etiology, clinical characteristics, and outcomes of meningitis among HIV-infected adults.* Methods.* A prospective cross-sectional hospital based study was conducted among HIV-infected patients aged ≥18 years admitted to the medical wards with symptoms and signs of meningitis. Sociodemographic and clinical information were collected using a standardized data collection tool. Lumbar puncture was performed to all patients; cerebrospinal fluid samples were sent for analysis.* Results.* Among 60 HIV-infected adults clinically diagnosed to have meningitis, 55 had CSF profiles consistent with meningitis. Of these, 14 (25.5%) had a laboratory-confirmed etiology while 41 (74.5%) had no isolate identified.* Cryptococcus neoformans* was the commonest cause of meningitis occurring in 11 (18.3%) of patients followed by* Mycobacterium tuberculosis* (6.7%). The in-hospital mortality was 20/55 (36.4%). Independent predictors of mortality were low baseline CD4 count and turbid CSF appearance.* Conclusion.* Cryptococcal meningitis is the most prevalent laboratory-confirmed etiological agent among adult HIV-infected patients with suspected meningitis admitted to medical wards in Western Tanzania. Mortality rate in this population remains unacceptably high. Improving diagnostic capacity and early treatment may help to decrease the mortality rate.

## 1. Introduction

Meningitis is common and is among the most serious infections occurring in individuals with HIV infection [1, 2]. The etiology of meningitis in HIV-endemic areas has been significantly altered in favor of cryptococcal meningitis and TB meningitis [1, 3, 4]. Evidence shows that cryptococcal meningitis is the most common fungal meningitis and an important opportunistic infection in patients living with HIV [2, 5–8]. TB meningitis has also been shown to be the second most common cause of meningitis, with a case fatality rate of up to 67% among HIV-infected individuals [4, 9, 10].

The symptoms of meningitis among HIV-infected individuals may vary depending on the cause. In one study done in Durban, South Africa, among HIV patients coinfected with tuberculosis (TB), the most common combination of presenting clinical features for patients suspected to have meningitis was headache and neck stiffness, seen in 78.6% of patients. The triad of headache, vomiting, and neck stiffness was observed in 35.7% whereas only 1% of patients presented with fever [3]. In another study done in Zimbabwe, patients with pyogenic meningitis presented with fever and marked alteration in mental status, while the main presenting feature of cryptococcal meningitis was headache. Patients with TB tended to be admitted with marked mental impairment, compared with that seen with other forms of meningitis [4].

The diagnosis of certain types of meningitis, particularly TB meningitis, is often difficult in resource-limited settings due to the low sensitivity of standard smear for acid-fast bacilli (AFB), which is often the only diagnostic assay available. Currently, the true prevalence of specific microbiological etiologies, clinical characteristics, and outcomes of meningitis in our setting are not well defined.

In order to optimize patient care, the epidemiologic trends of meningitis among HIV-infected patients should be examined frequently because any change may influence the choice of initial empiric antibiotics. Therefore, it was the aim of this study to determine the laboratory-confirmed etiological agents, clinical characteristics, and outcomes of meningitis among HIV-infected adults admitted to the medical wards of Bugando Medical Centre with symptoms and signs of meningitis.

## 2. Materials and Methods

### 2.1. Study Design and Study Area

This was a cross-sectional study with prospective follow-up to determine in-hospital mortality/outcomes involving all HIV-infected adult patients ≥18 years old admitted to the medical wards of BMC for symptoms and signs of meningitis. BMC is the referral hospital for 13 million people in northwest Tanzania. It is located on the shore of Lake Victoria where the prevalence of HIV is over 6%.

### 2.2. Patient Population

We serially enrolled all HIV-infected patients aged ≥18 years who were admitted to BMC medical wards with clinical suspicion of meningitis over a six-month period. Critically ill patients, patients with focal neurological signs or papilloedema, and those who had been treated for meningitis in the past one month were excluded.

### 2.3. Data Collection and Laboratory Analyses

All HIV-infected adults ≥18 years old admitted to the medical wards of BMC during the study period were screened for symptoms and signs of meningitis. Patients (or their caretakers) were interviewed using a standardized data collection tool/structured questionnaire to collect demographic information, clinical symptoms, and other information. Then, patients were examined within 24 hours of admission to determine the presence or absence of physical signs of meningitis. Patients with lateralizing signs and/or papilloedema on fundoscopy were excluded from the study because lumbar punctures would be contraindicated given our inability to rule out a space-occupying lesion with a computed tomography scan. For all enrolled patients, laboratory investigations including bedside lumbar puncture were taken for assessment. CSF analysis included biochemistry, cytology, Gram stain, AFB smear using Ziehl-Neelsen stain, TB culture, and India ink stain. Cryptococcal antigen test (CrAg) was performed using latest agglutination test kit (CALAS, Meridian Bioscience Europe, Nice, France) according to the manufacturer's instructions. Other laboratory investigations included full blood picture, erythrocyte sedimentation rate (ESR), and CD4^+^ T-cell (CD4) count. The CD4 counts were performed using Automated FACS Calibur Flow Cytometry machine (BD, San Jose, USA). All results were provided to clinicians who made all decisions about treatment for meningitis and other conditions. All patients with clinical meningitis were followed up for at least 2 weeks or until the time of discharge. Patients discharged prior to 2 weeks from study enrollment were contacted at 2 weeks to determine outcomes.

To test for cryptococcal meningitis, all HIV-infected patients had an additional 5 milliliters of blood collected for serum cryptococcal antigen assay. After lumbar puncture, about 4 milliliters of CSF was sent for both cryptococcal antigen and India ink staining. Testing of both serum and CSF for CrAg relied on the cryptococcal antigen assay (Immuno-Mycologics Inc. (IMMY), Oklahoma, USA). This assay is a World Health Organization (WHO) approved test that is used routinely for diagnosis of cryptococcal meningitis at BMC.

Other bacteria were identified by Gram staining of centrifuged sediment from CSF and were isolated using bacterial culture on blood and chocolate agars for 72 hours.

### 2.4. Statistical Analysis

Data were double entered into Microsoft Excel and analyzed using Stata version 11 (College Station, Texas). Continuous variables were presented as means (standard deviation (SD)) or medians (interquartile range (IQR)), and binary variables were presented as proportions (%). We analyzed clinical characteristics associated with various meningitis syndromes using the Wilcoxon rank-sum and Chi-squared or Fisher's Exact tests where appropriate. Associations between variables were considered to be significant if the *p* value was <0.05 at 95% CI.

### 2.5. Ethical Issues

Permission to conduct the study was sought from the Joint Research and Publication Committee of CUHAS/BMC. Patients were only involved after both verbal and written consent. Lumbar puncture was performed by the principal investigator, observing aseptic technique, ensuring minimal trauma to patients. All CSF analyses were done as per existing hospital guidelines; all patients found to have meningitis were treated as per existing hospital guidelines.

## 3. Results

### 3.1. Baseline Characteristics of Patients Enrolled in the Study

During the six-month study period from November 2012 to May 2013, a total of 1971 adults were admitted to BMC medical wards. Out of these, 73 were HIV-infected and had features of meningitis. Four patients refused consent, five had papilloedema, and the other four had traumatic unsuccessful lumbar punctures. Therefore, 60 patients were enrolled in the study. The median age of the study population was 39 years (interquartile range [IQR 32.5–47] (Table 1)). The majority (41, 68.3%) were females. One-half of the patients had unknown HIV status on admission. The median baseline CD4 count was 89 cells/uL [IQR 31–226]. About one-third of the study population (18, 30%) was on antiretroviral therapy (ART) on admission and the median duration on ART was 16 months [IQR 3–32]. Of the four classic symptoms of meningitis, 45 (75%) had headache and neck stiffness, 34 (56.7%) had three symptoms (headache, fever, and neck stiffness), and 24 (40%) had all four symptoms.

### 3.2. Admission Lumbar Puncture Findings

The median lumbar puncture CSF opening pressure was 18 cm of H_2_O [IQR 11–25], and 26 patients (43.3%) had elevated opening pressures (above 20 cm of H_2_O) (Table 2). The CSF of the majority (38, 63.3%) appeared clear in color. Cryptococcal antigen was positive in both serum and CSF of 11 patients (18.3%) and India ink showed encapsulated yeast cells in 8 of these 11 patients (72.7%). No patient had a positive India ink with a negative CSF CrAg test. The median CSF white blood cell (WBC) count was 19.5 cells/mm^3^ [IQR 6–53.5] and the majority (37, 61.7%) had a CSF lymphocytic predominance with more than 50% of WBC in CSF classified as lymphocytes.

Only 3 (5%) of all enrolled patients had combination of all abnormal signs/features on lumbar puncture (high lumbar puncture opening pressure, elevated CSF protein, elevated CSF WBC, and turbid CSF appearance). Among the 11 patients with cryptococcal antigen test positive, 10 (90.9%) of them had elevated CSF WBC (>5 cells/mm^3^). However, all 8 patients with India ink positive had combination of three of the four signs mentioned above.

The CSF Gram stain was positive in 5 (8.3%) and CSF routine culture was positive in 5 patients (8.3%). Four of five had encapsulated yeast on Gram stain and culture, and the fifth had Gram-positive cocci in pairs confirmed as* S. pneumoniae* on culture. Stain was positive in three (5%), whereas CSF TB culture was positive in these three, as well as in one additional patient, for a total of 4 (6.7%).

### 3.3. Prevalence of Laboratory-Confirmed Meningitis

The standard microbiological analysis of CSF was done in all 60 patients. Cryptococcal meningitis was confirmed through CSF CrAg in 11 patients (18.3%) with the India ink being positive in 8 of 11 (13.3% of total patients or 72.7% of confirmed cryptococcal cases). Tuberculous meningitis was confirmed in 4 patients (6.7%). Three of four culture-confirmed cases diagnosed in the laboratory were AFB smear-positive. Among patients with cryptococcal meningitis, 2 (18.2%) of them were coinfected with tuberculous meningitis (TBM).

Other bacterial meningitis was confirmed in only 1 patient (1.7%), who had* Streptococcus pneumonia*. The majority of patients had aseptic meningitis (41, 68.3%) with abnormal findings in the CSF (i.e., they had elevated WBC > 5 cells/mm^3^ on CSF but no bacteria or fungi on microscopy or culture). The other 5 patients (8.3%) had sterile CSF with normal WBC counts and, hence, were regarded as not having meningitis.

### 3.4. Predictors of Mortality for Meningitis

Low recent CD4 count was a significant predictor of mortality among this population, with patients who died having a median current CD4 count of 50 [15–100], compared with 98 [46–338] in those who did not die (*p* = 0.01). Also turbid CSF was found to be a significant predictor of mortality among these patients (*p* value = 0.001) (Table 3).

## 4. Discussion

This was the first cross-sectional study to determine different etiologies of meningitis among adult HIV-infected patients admitted at BMC. Among 60 adult HIV-infected patients enrolled in this study, 14 (23%) of them had confirmed microbiological etiologies and 41 (68%) others had no defined isolates in the CSF though they had laboratory findings (elevated CSF WBC) which were suggestive of meningitis. Our study also supports findings of other studies from sub-Saharan Africa that have shown* Cryptococcus* followed by tuberculosis is one of the leading causes of meningitis diagnosed in this population [3, 9]. Low recent CD4 count and turbid CSF were significantly associated with mortality from meningitis in our study population.

As described in two previous studies done at our hospital (BMC), cryptococcal meningitis remains a common cause of meningitis and death, among adult HIV/AIDS patients in our setting [6, 11]. This finding is also in keeping with studies from Muhimbili National Hospital and another study done in Durban, South Africa [2, 3]. In our study, the sensitivity of India ink in the diagnosing CM was high (72.7%) compared to previous studies. This could be explained by the fact that our patients were ART-naïve, and hence they may have had high fungal burdens and therefore a high positivity India ink CSF stains, consistent with other studies [6, 9, 12, 13].

Among patients with culture-proven TB meningitis, 75% had positive AFB smear; this is high sensitivity of smear microscopy compared to a study in Cape Town, South Africa, where a very low level of 1.3% was observed [9]. The high sensitivity of the AFB smear of 75% observed in our study could be due to the use of large volume of CSF (10 mLs); this confirmed what was described previously in Vietnam whereby the use of large volume CSF increased the diagnostic yield for TBM from <50% to 69% [14]. Our study finding concurs with another study done in the Philippines whereby only 4/91 (4.4%) patients with chronic meningitis had confirmed TBM by culture or AFB smear. However, in that study, the additional use of another test (like basal meningeal enhancement on contrast CT scan of the head) demonstrated that 44 patients fulfilled the criteria of definite TBM [15]. The use of this test in our setting is not cost-effective. Taken together, this study and ours highlight the continued challenges of diagnosing tuberculous meningitis and the urgent need for improved diagnostic resources.

Diagnosis of other types of bacterial meningitis in our study may also have been complicated by the sterilizing effect of antibiotics in the CSF. Because antibiotics are readily available at any local pharmacy in our setting, clinical experience suggests that most patients presenting to the hospital with fever have already tried several courses of antibiotics at home. This is evidenced by several recent studies that documented that most patients report the use of either prescribed or self-prescribed antibiotics prior to their admission to our hospital [16, 17]. We suspect that some of the cases classified as aseptic meningitis may have been partially treated bacterial meningitis whose CSF no longer had viable bacteria.

It was observed that, among HIV-infected patients with meningitis in this study, 36.7% died within the first 2 weeks of hospital admission. This finding is slightly lower compared with one study done in South Africa in which meningitis was associated with poor outcomes including high mortality in about 40% of diagnosed patients [3]. Importantly, among our study patients with cryptococcal meningitis alone, the mortality rate is relatively lower at 36.4%. Though these numbers are small, our work suggests some improvement compared to previous studies done in our hospital setting which found mortality rates of 66%, which decreased to 43% after implementation of routine serial lumbar punctures for patients with cryptococcal meningitis [6, 11]. We have continued to use serial lumbar punctures, together with high-dose intravenous fluconazole, to manage patients admitted with cryptococcal meningitis to our hospital.

The very high 75% mortality rate for patients with TBM draws attention to the continued need for better diagnosis and treatment. Of 3 deaths, 2 had AFB smear-positive, suggesting a high burden of mycobacteria. This high mortality rate among patients with TBM is supported by another larger study done in South Africa where the mortality rate was about 66.7% [3].

## 5. Conclusion

Cryptococcal meningitis is the most prevalent laboratory-confirmed isolate among adult HIV-infected patients admitted with features suggestive of meningitis to the BMC medical wards. Tuberculous meningitis is the second most common cause of confirmed meningitis; however, it remains difficult to diagnose due to challenges with diagnostic tools and likely has a higher prevalence in reality. Our work calls attention to the ongoing need for improved diagnosis and management for patients presenting with meningitis in resource-limited settings.

## Figures and Tables

**Table 1:** Baseline demographic and clinical characteristics of HIV-infected adults admitted with features of meningitis to the medical wards of Bugando Medical Centre between November 2012 and May 2013 (*n* = 60).

Characteristic	Value median (IQR) or number (%)
*Age in years*	39 (32.5–47)

*Female gender *	41 (68.3%)

*Marital status*	
Single	9 (15%)
Married	31 (51.7%)
Separated/divorced/widowed	20 (33.3%)

*Urban area of residence*	43 (71.7%)

*Occupation*	
Unemployed/self-employed	54 (90%)
Employed	6 (10%)

*Body mass index (kg/m* ^*2*^)	20.6 (19.1–22.4)

*Symptoms and signs on admission*	
Headache	60 (100%)
Duration of headache (days)	8 (7–14)
Vomiting	38 (63.3%)
Duration of vomiting (days)	2.5 (2–5)
History of weight loss	34 (56.7%)
Fever documented at the time of admission	49 (81.7%)
Altered mental status	33 (55.0%)
Neck stiffness	45 (75.0%)
Photophobia	25 (41.7%)
Kerning's sign positive	26 (43.3%)
Brudzinski sign positive	15 (25.0%)

*HIV status on admission*	
Known	30 (50%)
New diagnosis	30 (50%)

*Baseline CD4 count (cells/uL)*	89 (31–226)

*Nadir CD4 count (cells/uL) in known HIV-infected patients*	55 (15–225)

*Most recent CD4 count (cells/uL) in known HIV-infected patients*	92.5 (35.5–260.5)

*Currently receiving ART*	18 (30%)

*Duration on ART (months) *	16 [3–32]

*Peripheral blood WBC (×10* ^*3*^ */uL)*	5.6 (4.9–7.3)

*Peripheral blood WBC differential*	
Neutrophils (%)	67.6 (52.5–73.8)
Lymphocytes (%)	23.2 (18.2–33.6)
Monocytes (%)	6.7 (4.3–8.3)

*Hemoglobin level (g/dL)*	11.1 (7.85–12.3)

*ESR (mm/1st hour)*	45 (35–65)

**Table 2:** Admission lumbar puncture findings in HIV-infected adults admitted with features of meningitis to the medical wards of Bugando Medical Centre between November 2012 and May 2013 (*n* = 60).

Characteristic	Value/median (%)/(IQR)
*Lumbar puncture CSF opening pressure (cm of H*_*2*_ *O)*	18 (11–25)
Normal opening pressure (<20 cm of H_2_O)	34 (56.6%)
Elevated opening pressure (≥20 cm of H_2_O)	26 (43.3%)

*CSF appearance*	
Clear	38 (63.3%)

*Cryptococcal antigen positive*	
Serum	11 (18.3%)
CSF	11 (18.3%)

*CSF India ink positive*	8 (13.3%)

*CSF WBC count (cells/mm* ^*3*^)	19.5 (6–53.5)

*CSFWBC differential predominance*	
Lymphocytes	37 (61.7%)
Neutrophils	6 (10.0%)
Monocytes	17 (28.3%)

*CSF protein (Pandy's test) positive*	41 (68.3%)

*CSF Gram stain positive*	5 (8.3%)
Gram-positive cocci in pairs	1 (1.7%)
Encapsulated yeast cells	4 (6.7%)

*CSF routine culture positive*	5 (8.3%)

*CSF routine culture isolates*	
* Streptococcus pneumoniae*	1 (1.7%)
* Cryptococcus neoformans*	4 (6.7%)

*CSF AFB ZN stain positive*	3 (5.0%)

*CSF TB culture positive*	4 (6.7%)

**Table 3:** Clinical predictors of mortality among 60 HIV-infected adults patients admitted with meningitis to medical wards of Bugando Medical Centre, Mwanza.

Predictive factor (variable)	Meningitis patients		*p* value
	Death (*n* = 22) number (%) or median (IQR)	Alive (*n* = 38) number (%) or median (IQR)	
*Age in years*	40 [32–46]	39 [33–47]	0.55

*Gender *			
Male	8 (42.1)	11 (57.9)	0.376
Female	14 (34.1)	27 (65.9)	

*Marital status*			
Married	10 (32.3)	21 (67.7)	0.321
Not married	12 (41.4)	17 (58.6)	

*Occupation*			
Unemployed	19 (35.2)	35 (64.8)	0.384
Employed	3 (50)	3 (50)	

*Residence*			
Rural	4 (23.5)	13 (76.5)	0.151
Urban	18 (41.9)	25 (58.1)	

*Duration of headache*			
≤14 (days)	18 (37.5)	30 (62.5)	0.534
>14 (days)	4 (33.3)	8 (66.7)	

*Vomiting *			
Yes	14 (36.8)	24 (63.2)	0.597
No	8 (36.4)	14 (63.6)	

*Duration of vomiting *			
≤7 days	12 (33.3)	24 (66.7)	0.129
>7 days	2 (100)	0	

*Fever *			
Yes	17 (34.7)	32 (65.3)	0.367
No	5 (45.5)	6 (54.5)	

*Altered mental status *			
Yes	15 (45.5)	18 (54.5)	0.098
No	7 (25.9)	20 (74.1)	

*Neck stiffness*			
Yes	18 (40)	27 (60)	0.272
No	4 (26.7)	11 (73.3)	

*Photophobia *			
Yes	6 (24)	19 (76)	0.073
No	16 (45.7)	19 (54.3)	

*Kerning's sign*			
Yes	12 (46.2)	14 (53.8)	0.144
No	10 (29.4)	24 (70.6)	

*Brudzinski sign*			
Yes	8 (53.3)	7 (46.7)	0.109
No	14 (31.1)	31 (68.9)	

*Baseline CD4 count* (cells/uL)	50 [15–100]	98 [46–338]	**0.01**

*Recent CD4 count* (cells/uL)	59.5 [30–132]	100 [46–338]	0.08

*On ART *			
Yes	5 (27.8)	1 3 (72.2)	0.263
No	17 (40.5)	25 (59.5)	

*Duration on ART*	32 [10–83]	14 [3–27]	0.29

*LP opening pressure*			
<20 cm H_2_O	11 (32.4)	23 (67.6)	0.300
≥20 cm H_2_O	11 (42.3)	15 (57.7)	

*CSF appearance *			
Turbid	20 (90.9)	2 (9.1)	**<0.001**
Nonturbid/clear	2 (5.2)	36 (94.8)	

*India ink*			
Yes	3 (37.5)	5 (62.5)	0.623
No	19 (36.5)	33 (63.5)	

*Etiology confirmed meningitis*			
Yes	7 (50)	7 (50)	0.192
No	15 (32.6)	31 (67.4)	
